# Trends in measles cases in Bayelsa state, Nigeria: a five-year review of case-based surveillance data (2014–2018)

**DOI:** 10.1186/s12889-020-09070-0

**Published:** 2020-06-15

**Authors:** Neni Aworabhi-Oki, T. Numbere, M. S. Balogun, A. Usman, R. Utulu, N. Ebere, W. Omubo, J. Stow, S. Abba, A. Olorukooba

**Affiliations:** 1 Nigeria Field Epidemiology and Laboratory Training Program, Asokoro, Abuja, Nigeria; 2 Bayelsa State Ministry of Health, Yenagoa, Nigeria; 3 Ahmadu Bello University, Zaria, Nigeria

**Keywords:** Measles, Surveillance, Vaccination, Nigeria, Trend, Predictors

## Abstract

**Background:**

Measles is a vaccine preventable, highly transmissible viral infection that affects mostly children under five years. It has been ear marked for elimination and Nigeria adopted the measles elimination strategies of the World Health Organization (WHO) African region to reduce cases and deaths. This study was done to determine trends in measles cases in Bayelsa state, to describe cases in terms of person and place, identify gaps in the case-based surveillance data collection system and identify risk factors for measles infection.

**Methods:**

We carried out a secondary data analysis of measles case-based surveillance data for the period of January 2014 to December 2018 obtained in Microsoft Excel from the State Ministry of Health. Cases were defined according to WHO standard case definitions. We calculated frequencies, proportions, estimated odds ratios (OR), 95% confidence intervals (CI) and multivariate analysis.

**Results:**

A total of 449 cases of measles were reported. There were 245(54.6%) males and the most affected age group was 1–4 years with 288(64.1%) cases. Of all cases, 289(9.35%) were confirmed and 70 (48.27%) had received at least one dose of measles vaccine. There was an all-year transmission with increased cases in the 4th quarter of the year. Yenegoa local government area had the highest number of cases. Timeliness of specimen reaching the laboratory and the proportion of specimens received at the laboratory with results sent to the national level timely were below WHO recommended 80% respectively. Predictors of measles infection were, age less than 5 years (AOR: 0.57, 95% CI: 0.36–0.91) and residing in an urban area (AOR: 1.55, 95% CI:1.02–2.34).

**Conclusions:**

Measles infection occurred all-year round, with children less than 5 years being more affected. Measles case-based surveillance system showed high levels of case investigation with poor data quality and poor but improving indicators. Being less than 5 years was protective of measles while living in urban areas increased risk for infection. We recommended to the state government to prioritize immunization activities in the urban centers, start campaigns by the 4th quarter and continue to support measles surveillance activities and the federal government to strengthen regional laboratory capacities.

## Background

Measles is a vaccine-preventable, highly transmissible viral infection that affects mostly children under five years [1]. It is a cause of mortality and morbidity world-wide [2]. Despite achieving and sustaining global measles vaccination coverage of about 80% over the past decade, measles remains the fifth leading cause of mortality among children aged less than 5 years [3]. It accounts for more than 30 million cases annually and 0.9 million deaths every year, with approximately half of these occurring in Africa [3].

Measles is transmission mainly occurs at the end of the rainy season and epidemics tend to be seen in the dry season (February, March and April) [1].It is considered a potentially eliminable disease because the reservoir is exclusively human, also sensitive and specific diagnostic tests, as well as safe effective vaccines are available [4]. Thus it has been targeted for eradication [5]. However, due to social and political factors and high transmissibility, elimination has been achieved in only few areas of the world [6, 7]. To interrupt measles transmission, 95% vaccination coverage is required. Failure to deliver at least one dose of measles vaccine to all infants remains the main reason for high measles morbidity and mortality [2]. From 2000 to 2017, measles vaccination prevented an estimated 21.1 million deaths [8].

Measles control activities in World Health Organization (WHO) African Region aim to reduce measles deaths [2]. The strategies implemented include improving routine vaccination coverage, providing a second opportunity for measles vaccination through supplementary immunization activities (SIAs), improving measles case management, conducting case-based measles surveillance to enable immunization strategies to be properly adjusted and to document the decline in cases and progress in eliminating the disease, supported by the measles laboratory network [2].

Data generated from public health surveillance systems is used for guiding immediate public health action, for program planning and evaluation, to monitor trends in the burden of disease and formulating research hypotheses [3]. Measles case-based surveillance is a system put in place to detect cases and outbreaks of measles. It involves reporting and investigating any suspected case of measles, to use the data to evaluate immunization efforts and predict outbreaks through the identification of geographical areas and age-groups at risk [9].

It has been reported that Nigeria has a measles vaccine coverage of less than 50% [1]. Despite conducting measles campaigns in 2017 in the northern states and in 2018 for the southern states, outbreaks were still reported from different states in the country [10]. As of August 2019, Nigeria had reported 30,457 suspected cases to the WHO of which 24,994 were confirmed [8]. This was the second highest reported number of cases among member states for the period. In 2006, measles case-based surveillance became operational in Nigeria using the resources and infrastructure of the already established surveillance for acute flaccid paralysis [11]. and Integrated Disease Surveillance and Response (IDSR) system.

Bayelsa State, located in the southern region of Nigeria had measles vaccination coverage of 41.8% for children 12–23 months with any evidence of vaccination in 2016 [12]. A measles outbreak was reported in Southern Ijaw local government area (LGA) in 2010 [13]. A measles mass vaccination campaign was conducted in the state in 2018 targeting children 9 months to 59 months; however, cases were still reported from all LGAs [10, 14]. From the National Demographic Health Survey 2018, Measles vaccination coverage was 71.3% while administrative coverage from the District Health Information Software 2 (DHIS 2) was 56% in 2018.

Determining the trend of cases will highlight immunization efforts, help in outbreak prevention through the recognition of geographical areas and age-groups likely to be affected and also highlight progress towards attaining measles elimination goal in the state.

This study was carried out to determine trends in measles cases in the state, to describe cases in terms of person and place, identify gaps in the case-based surveillance data collection system and identify risk factors for measles infection.

## Methods

### Study area

Bayelsa State is located in the South-South geopolitical zone of Nigeria. It is in the tropical rainforest. The mean monthly temperature is in the range of 25 °C to 31 °C.The mean annual temperature is uniform for the entire state. The hottest months are December to April. Relative humidity is high in the state throughout the year and decreases slightly in the dry season [15]. The projected population for 2018 was 2,332,787 and population under-five years was 494,310 from 2006 population census [16]. The State has a total of 211 health facilities offering routine immunization services distributed across 8 LGAs. The state has an area of around 21′000 square kms and about three quarters of its total area lies under water [17]. Three Supplemental Immunization activities (SIAs) was conducted between 2014 and 2018 with the target age for each between 9 months to 59 months. The State commenced Measles second dose in 2019.

Measles reporting through the IDSR system is adopted in Bayelsa State. Information flows from the health facility focal persons, to the LGA disease surveillance and notification officers (DSNOs), to the State DSNO, and then to the Federal Ministry of Health and Nigeria Center for Disease Control. Feedback goes through the reverse direction. A case investigation form is completed for each suspected case and blood sample is collected. The blood sample is then sent to a WHO-accredited laboratory for confirmation [18].

### Study design

We carried out a secondary data analysis of measles case-based surveillance data for Bayelsa State for the period of January 2014 to December 2018.

### Data source

Data on Measles case-based surveillance and laboratory results from 2014 to 2018 was obtained from the Bayelsa State Ministry of Health.

Cases were defined according the WHO standard case definitions as follows:

A suspected case: is the occurrence of fever and a maculopapular rash with any one of cough, coryza, or conjunctivitis or any illness in a person that a clinician suspects to be measles [2].

A confirmed measles case: is classified as either laboratory-confirmed, epidemiologically linked, or clinically compatible [2].

Laboratory confirmed case: when measles-specific IgM antibody is detected in serum by enzyme-linked immunosorbent assay (ELISA) in the absence of measles vaccination within 30 days before specimen collection [2].

Epidemiological linkage: when a suspect measles case has had contact with or lives in the same locality as a person with laboratory-confirmed measles infection [2].

Clinically compatible: those cases which satisfy the clinical case definition, but a laboratory test or an epidemiological link is lacking [2].

### Data management

Data was retrieved in Microsoft Excel. Relevant variables were sorted, extracted and cleaned from the surveillance data. The variables were age, sex, number of cases, reporting district, date of onset, vaccine doses received, date sample was collected and sent to laboratory, laboratory results and date result was received.

Using Microsoft Excel 2016 and Epi Info version 7.0, we calculated frequencies, proportions, estimated odds ratios (OR) and 95% confidence intervals (CI) to identify risk factors for measles infection in the state. Multivariate analysis was done to identify independent risk factors for measles infection.

### Ethical consideration

Ethical approval with approval number BSHREC/Vol. 1/19/6 was obtained to use the data from the Public Health Department of the Bayelsa State Ministry of Health. We maintained confidentiality of subjects by excluding all identifying information such as name and address from the analysis. Data was stored in a separate folder on a passworded computer that was only accessible by the researcher.

## Results

A total of 449 cases of measles were reported between January 2014 and December 2018. There were 245 (54.6%) males, the most number of cases occurred in the age-group 1–4 years (64.1%), followed by children aged between 5 and 14 years (18.3%) (Table 1).

**Table 1:** Socio-demographic characteristics of reported measles cases in Bayelsa State, 2014–2018

Variables	No. of suspected cases n (%)	No. of confirmed cases n (%)
**Sex**		
**Females**	204 (45.4)	134 (46.4)
**Males**	245 (54.6)	155 (53.6)
**Age group (years)**		
**< 1**	59 (13.1)	40 (13.8)
**1–4**	288 (64.1)	173 (59.9)
**5–14**	82 (18.3)	59 (20.4)
**≥ 15**	20 (4.5)	17 (5.9)
**Place of Residence**		
**Urban**	168	118
**Rural**	281	171
**Total**	**449**	**289**

**Fig. 1:**
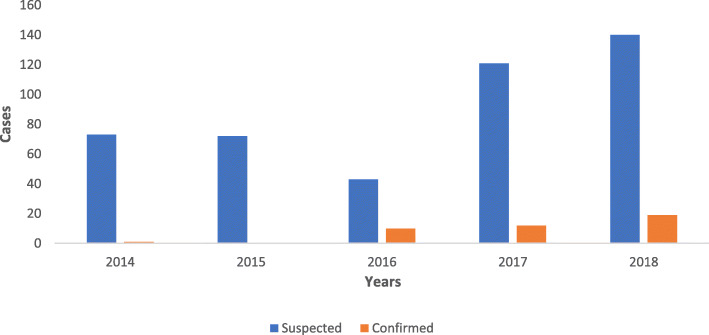
Suspected measles cases and laboratory confirmed measles cases in Bayelsa State from 2014 to 2018

Of the 449 cases recorded within the five-year period, 289 (9.35%) were confirmed (Table 1) by laboratory diagnosis, epidemiological linkage and clinical compatibility (Table 2). The highest proportion of laboratory confirmed cases in a year was 19 (45.23%) in 2018. No laboratory confirmed cases were recorded in 2015 (Fig. 1).

**Table 2:** Confirmed Measles cases in Bayelsa State, 2014–2018

S/N	Category	No. of cases
1.	Laboratory confirmed	42
2.	Epidemiologic Linkage	245
3.	Clinically compatible	2
	**Total**	**289**

**Fig. 2:**
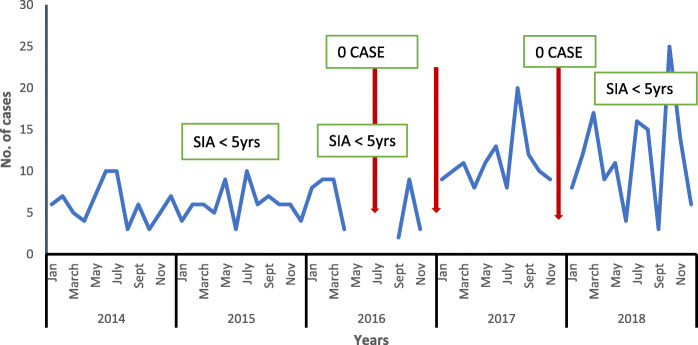
Trend of measles cases in Bayelsa State, 2014–2018

**Fig. 3:**
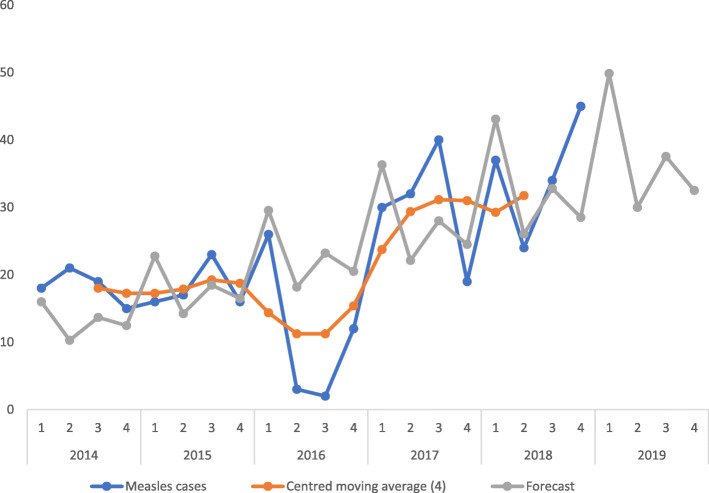
Time series forecast model for measles cases in Bayelsa State (2014–2019)

There was an all-year transmission, with peaks in May and July. Reports for some months were not available (Fig. 2), for 2016 completeness of reporting was 58.3% while in 2017 it was 91.6% (Table 4). The time trend series analysis forecasted expected number of cases for quarters 1–4 in 2019 as 49,30,37 and 32 respectively. There was a rise in cases in the 4th quarter of each year as well as in the forecast for 2019, however the model was only slightly significant (Fig. 3).

**Fig. 4:**
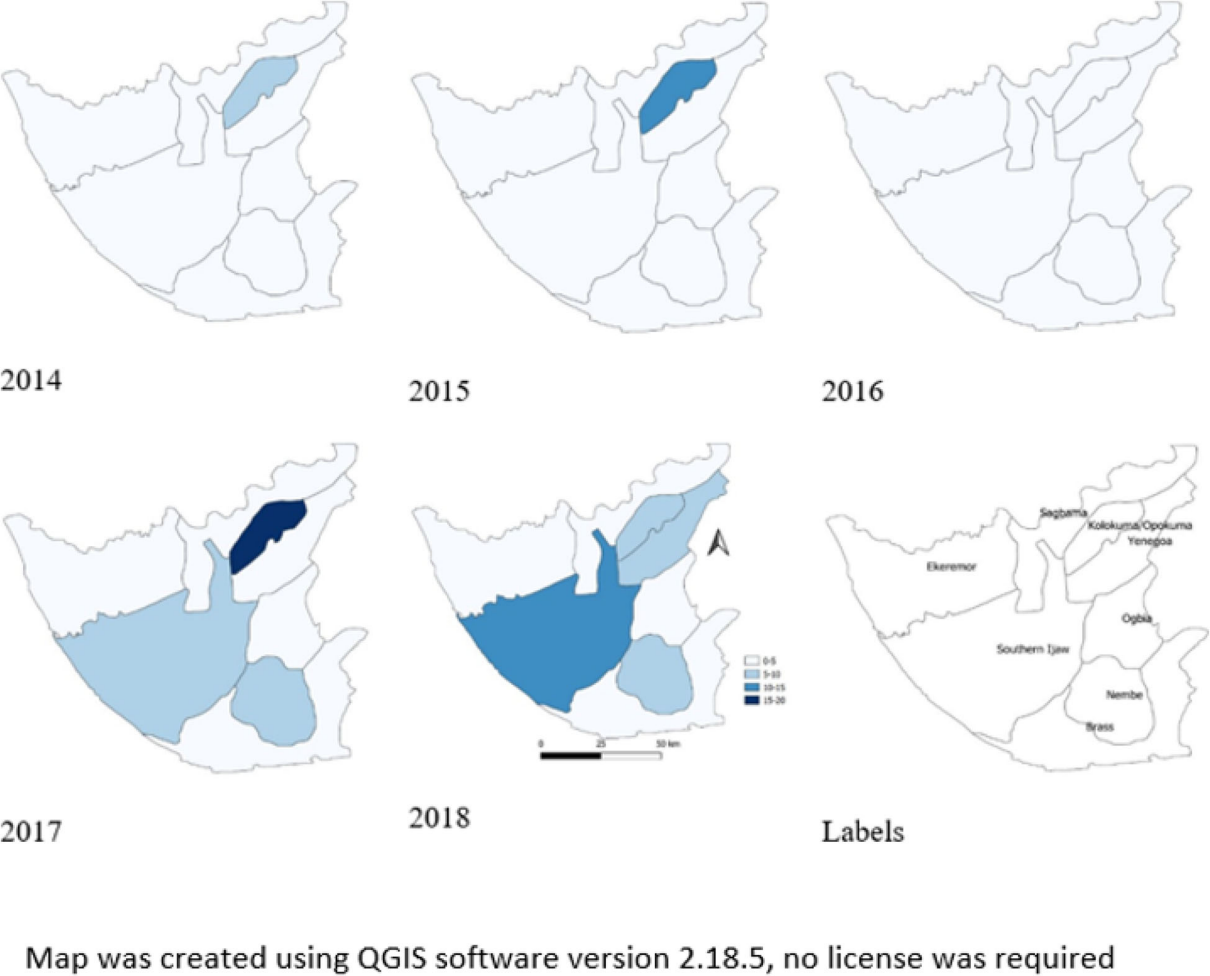
Geographical distribution of measles cases by incidence rate per 100,000 population for LGAs in Bayelsa State from 2014 to 2018

Yenegoa LGA had the highest number of reported cases within the five-year period; however, incidence rate per 100,000 was high in Kolokuma/Opokuma, Nembe, Yenegoa (Urban area) and Southern Ijaw LGA over the five years under review (Fig. 4).

Data on the number of vaccine doses received was not collected for all reported cases; only 145 (32.29%) of all cases had that information, of which only 70 (48.27%) had received at least one dose of measles vaccine and more than half (51.72%) of the cases had not received any measles vaccine at all (Table 3). However, 304 cases had missing information on vaccination status.

**Table 3:** Measles vaccine doses received in Bayelsa State from 2014 to 2018

No. of vaccine doses	Frequency	%
0	75	51.7
1	62	42.8
2	8	5.5
**Total**	**145**	**100**

There is an improvement in majority of the indicators over the years (Table 4), Non-Measles Febrile Rash illness and Timeliness of case investigation within 3 days was not calculated for 2016, 2017 and 2018 as the data was not available.

**Table 4:** Measles case-based surveillance indicators for Bayelsa State, 2014–2018

Measles Surveillance Indicators	Target	2014	2015	2016	2017	2018
Non-Measles Febrile Rash Illness Rate	At least 2 per 100,000 population	1.2	1.7	0	0	0
Proportion of districts that have reported at least 1 suspected case of measles with a blood specimen per year	At least 80%	100%	100%	100%	100%	100%
Incidence rate of confirmed measles		15.4	15.4	18.9	22.7	53.1
Proportion of Specimen reaching Laboratory in good condition	At least 90%	96%	96%	100%	100%	100%
Timeliness of case investigation within 3 days	> 80%	84.7%	73%	0	0	0
Completeness of reporting		58.3%	91.6%	100%	100%	100%

**Fig. 5:**
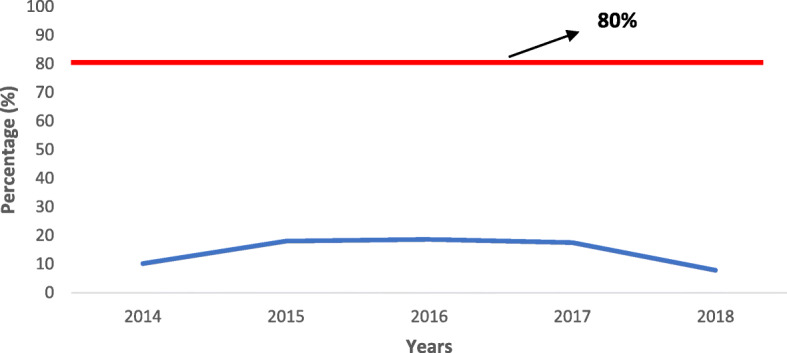
Proportion of measles specimen arriving the laboratory within 3 days of specimen collection

Timeliness of specimen reaching the laboratory within 3 days of specimen collection, was persistently low below the WHO recommended target of 80% for the five years (Fig. 5).

**Fig. 6:**
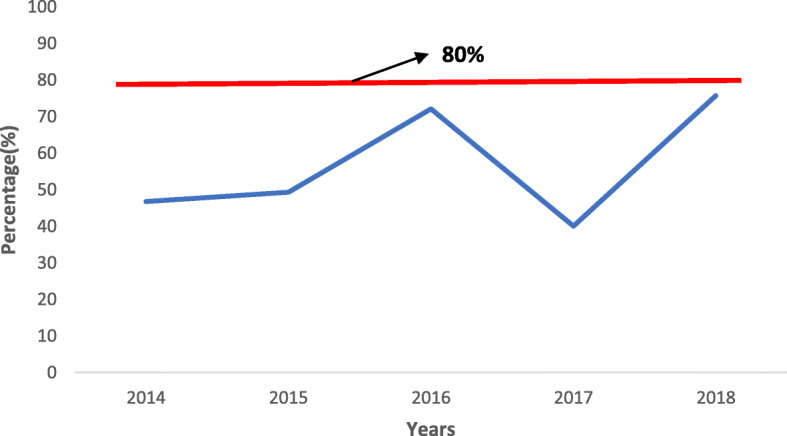
Proportion of feedback of serology results sent from the laboratory to the national level within 7 days of receipt of specimens, Bayelsa State, 2014–2018

The proportion of specimens received at the laboratory with results sent to the national level on time (within 7 days of specimen receipt at the laboratory) was below the recommended WHO target of 80% all through 2014–2018. However, a general increase in proportion of specimens with results sent out timely from the laboratory was noted in the period of 2015 and 2016, followed by a decline in 2017 and then an increase in 2018 (Fig. 6).

People greater than 5 years were significantly more likely to have measles infection compared to those less than five years, also the odds of having measles was significantly higher in those residing in the Urban areas than in the rural areas. The odds of having measles was higher among females although this association was not statistically significant (Table 5). People not vaccinated were more likely to have measles although this association was not statistically significant (Table 5).

**Table 5:** Exposure factors for Measles in Bayelsa State, 2014–2018

S/N	Variable	Frequency		OR	95% CI	AOR	95% CI
		Yes	No				
1	**Age (years)**						
	< 5	199	126	**0.57**	**0.36–0.90**	**0.57**	**0.36–0.91**
	≥ 5	91	33	1		**1**	
2	**Sex**						
	Male	156	89	0.91	0.62–1.35		
	Female	134	70	1			
3	**Place of residence**						
	Urban	118	49	**1.54**	**1.02–2.32**	**1.55**	**1.02–2.34**
	Rural	172	282	1		1	
4	**Vaccination Status**						
	None	36	39	1.03	0.53–1.98		
	At least one dose	33	37	1			

After controlling for age and sex, the logistic regression of exposure factors for measles showed that age less than 5 years remained a significant protective factor while residing in an urban area remained a significant exposure factor (Table 5).

## Discussion

This study, carried out to determine trends in measles cases and risk factors for measles in Bayelsa State found that the most reported age group were children less than 5 years, with the highest range between 1 and 4 years, most studies allude the highest prevalence of measles in under-five year children [19]. However, the analysis showed being less than five years as protective in this population and study period, this may be due to the small number of suspected cases reported over the period compared to the confirmed cases and the SIAs done during this period focusing on less than 5 years age group would have provided additional protection.

There was a preponderance of reported cases among males even though there is no known sex predilection as unvaccinated males and females are equally susceptible to infection by the measles virus [20]. However, our findings are consistent with that of studies that observed male preponderance of cases [19, 21]. However a study in Zimbabwe reported a similar ratio of males to females [3].

We noticed an all-year transmission of measles with two peaks in May and July in 2014,2015, 2017 and 2018 except in 2016 from May to August there were no reported cases due to health workers’ strike in the state. Similarly another study showed that measles cases were reported throughout the whole five-year period with no break in reporting, following similar patterns each year (from 2012 to 2016), though with a peak in number of reported cases in March [1]. This is different from what was reported in a study in Abia State where most of the cases of measles occurred in the dry season, with the peak in January and February [9]. However, it was recorded that measles transmission in Nigeria occurs through all months of the year, but peaks in the dry season (February, March and April) [1].

There was an increasing trend in the number of reported cases from 2014 to 2018 except in 2016 where a sharp decline was seen. This increase may be difficult to interpret as this may be attributed to either a low vaccination coverage or a strengthening surveillance system over the years. These options need to be explored. Further studies comparing vaccination coverage and surveillance activities during the period reviewed should explain this increase**.** Cases tend to start increasing at the end of the 3rd quarter of each year with a rise seen at the 4th quarter even in the 2019 forecast, this is expected as measles transmission is higher during hot seasons in Nigeria [1]. This finding is similar to that of a 10-year study conducted in Makurdi, Nigeria, it was discovered that the cases of measles in the Federal Medical Center was higher from the last to the first quarter in each successive year [22].

Only 9.35% of all reported measles cases were confirmed by laboratory diagnosis, this is quite low and is consistent with the findings in a similar study that found only 6.7% of measles cases in Nigeria over a five-year period were confirmed by laboratory testing [1]. This shows that laboratory confirmation of measles is still very low in the state. All suspected measles cases are expected to be confirmed by laboratory testing by so doing outbreaks will be detected promptly. This will also enable documentation of decline in cases and progress towards measles elimination [2].

The highest number of reported cases from Yenegoa LGA. may be attributable to the presence of a Federal Medical Centre in the LGA that serves as a referral center. In addition, being an urban area, the high population with overcrowding may predispose to measles infection. Measles being a highly contagious disease, recent contact and overcrowding are risk factors for disease transmission [21]. Ekeremor, a rural area and Sagbama had the least reported cases within the same period, this is different from what was observed in a similar study in Abia State where about 75% of the measles cases occurred in rural areas [9]. From this study, incidence rate was higher in urban areas and the odds of having measles was higher in those living in the urban areas than in the rural areas. A previous study revealed measles outbreak been less frequent in rural than in urban settings [23]. Another study carried out in Bayelsa State on community participation and childhood immunization coverage found that the immunization status of children in the rural community was significantly better than those in the urban community studied [24]. Implying that immunization in urban centers is inadequate.

Of the cases with data on vaccination status, 51.7% had not received any measles vaccine at all. This is not acceptable as free and effective measles vaccines were available in the state. From our study, people not vaccinated were more likely to have measles although this association was not statistically significant. Measles is vaccine-preventable and a child is eligible for measles vaccine once they attain nine months of age [2].

There was good sample collection, storage and transportation practices in the state. Indicators of performance of the surveillance system such as timeliness of specimen reaching the laboratory within 3 days of specimen collection, was persistently low below the WHO recommended target of 80% [2]. This delay in specimens reaching the laboratory could be a consequence of batching of specimens by the DSNOs for transportation to reference laboratories in Lagos where they are analyzed in order to save cost. This practice contributes to delay in confirmation of cases and outbreaks may be missed.

The proportion of feedback of serology results sent from the laboratory to the national level within 7 days of receipt of specimens was persistently low below the WHO recommended target of 80% [2], this is similar to findings in a study done in Zimbabwe [3]. However, a general increase was noted in the period of 2015 and 2016, followed by a decline in 2017 and then an increase in 2018. Timely feedback from the laboratory is important for confirmation of cases and prompt decision making. This also helps to boost the morale of surveillance officers who contribute to the functioning of the system.

The data obtained for this study had some limitations that restrict conclusions drawn from its analysis, and thus may limit generalization of the findings. The information was derived from reported cases in the surveillance system so there may have been unreported cases, however this data set will contain most cases in the state as the data was gotten from surveillance sites where most cases are likely to report. Many variables had missing data for several cases such as number of vaccine doses received (67% missing entries), date specimen was sent to the laboratory (4% missing entries) and date district received laboratory results (19% missing entries). Doubtful entries that produced 0 days for laboratory turn-around time and time interval between specimen collection and sent to the laboratory, these were excluded from the analysis. Mortality data was not collected and consequently case fatality rate could not be estimated. This is a pointer to gaps in data collection system in the state. Despite these limitations, this study was the first describing the measles trends in Bayelsa State and the accuracy of information on reported cases in the study depended on the accuracy of reports submitted and stored on the surveillance system. All available data over the study period was utilized.

## Conclusions

Measles infection occurred all-year round with increased cases in the 4th quarter of the year and with children less than 5 years being more affected. Measles case-based surveillance system for Bayelsa State showed high levels of case investigation with blood specimen collected. However, timeliness of transportation of specimens to the laboratory were often low with feedback of laboratory results improving over the years but data quality was poor with many missing variables. Being less than 5 years was protective of measles while living in urban areas was a significant risk factor. We recommended targeted SIAs in the state at the start of the 4th quarter and prioritization of immunization activities in the urban centers by the state government. The Federal Ministry of Health and Nigeria Center for Disease Control should strengthen regional laboratory capacities for prompt diagnosis of measles cases and improved laboratory turn-around time. The state government should support measles blood specimen transportation to reduce batching and conduct training and retraining of Disease Surveillance Notification Officers (DSNOs) and focal persons at regular intervals on data quality issues in collaboration with partners in the state.

## Data Availability

The datasets used and/or analyzed during the current study are available from the corresponding author on reasonable request.

## Abbreviations

WHO: World Health Organization
SIAs: Supplemental Immunization Activities
IDSR: Integrated Disease Surveillance and Response
LGAs: Local Government Areas
DSNOs: Disease Surveillance and Notification Officers
